# MALDI Imaging Mass Spectrometry Reveals Lipid Alterations in Physiological and Sertoli Cell-Only Syndrome Human Testicular Tissue Sections

**DOI:** 10.3390/ijms25158358

**Published:** 2024-07-31

**Authors:** Alexandra Sulc, Péter Czétány, Gábor Máté, András Balló, Dávid Semjén, Árpád Szántó, László Márk

**Affiliations:** 1 Department of Analytical Biochemistry, Institute of Biochemistry and Medical Chemistry, Medical School, University of Pécs, 7624 Pécs, Hungary; sulc.alexandra@pte.hu; 2 National Laboratory on Human Reproduction, University of Pécs, 7622 Pécs, Hungary; czetany.peter@pte.hu (P.C.); mate.gabor@pri.hu (G.M.); ballo.andras@pte.hu (A.B.); szanto.arpad@pte.hu (Á.S.); 3 Urology Clinic, University of Pécs, 7621 Pécs, Hungary; 4 Pannon Reproduction Institute, 8300 Tapolca, Hungary; 5 Institute of Pathology, Medical School, University of Pécs, 7624 Pécs, Hungary; semjen.david@pte.hu; 6 Imaging Centre for Life and Material Sciences, University of Pécs, 7624 Pécs, Hungary; 7 HUN-REN-PTE, Human Reproduction Research Group, 7624 Pécs, Hungary

**Keywords:** andrology, azoospermia, in vitro fertilization, lipidomics, imaging mass spectrometry, non-obstructive azoospermia, Sertoli cells, spermatogenesis, testis

## Abstract

Azoospermia, the absence of sperm cells in semen, affects around 15% of infertile males. Sertoli cell-only syndrome (SCOS) is the most common pathological lesion in the background of non-obstructive azoospermia and is characterised by the complete absence of germinal epithelium, with Sertoli cells exclusively present in the seminiferous tubules. Studies have shown a correlation between successful spermatogenesis and male fertility with lipid composition of spermatozoa, semen, seminal plasma or testis. The aim of this research was to discover the correlation between the Johnsen scoring system and phospholipid expressions in testicular cryosections of SCOS patients. MALDI imaging mass spectrometry is used to determine spatial distributions of molecular species, such as phospholipids. Phosphatidylcholines (PCs), phosphatidylethanolamines (PEs) and sphingomyelins (SMs) are the most abundant phospholipids in mammalian cells and testis. SMs, the structural components of plasma membranes, are crucial for spermatogenesis and sperm function. Plasmalogens, are unique PCs in testis with strong antioxidative properties. This study, using imaging mass spectrometry, demonstrates the local distribution of phospholipids, particularly SMs, PCs, plasmalogens and PEs in human testicular samples with SCOS for the first time. This study found a strong relationship between the Johnsen scoring system and phospholipid expression levels in human testicular tissues. Future findings could enable routine diagnostic techniques during microTESE procedures for successful sperm extraction.

## 1. Introduction

Spermatogenesis is the process of developing haploid spermatozoa from spermatogonia in seminiferous tubules. It starts with stem cell division, then mitosis, followed by meiosis. Primary spermatocytes divide into secondary spermatocytes, which further produce haploid spermatids, and finally transform into mature spermatozoa. Spermatogenesis occurs in testes within seminiferous tubules, with further sperm maturation happening in the ducts of the epididymistubules [1,2,3].

Spermatocytogenesis begins with a diploid spermatogonium deviating through mitosis, resulting in primary spermatocytes. Each primary spermatocyte migrates to the lumen, replicates DNA, and undergoes the first meiosis, producing secondary spermatocytes, which then divide into haploid sperm cells. During spermiogenesis, sperm cells form a tail, package DNA, and become dense and transcriptionally inactive [4,5].

Following this, the removal of excess cytoplasm and organelles occurs. This is the process known as spermiation, where Sertoli cells engulf the remnants. Intratesticular testosterone has a major impact on spermatogenesis via at least four critical processes: maintenance of blood-testis barrier, meiosis, Sertoli cell-spermatid adhesion, and sperm release into the lumen of the seminiferous tubules [6]. Spermatozoa transported to the epididymis gain motility and fertility. Sertoli cells provide structural and metabolic support, regulate tight junctions, and phagocytose residual cytoplasm. They also secrete substances for meiosis, testosterone concentration, and hormone regulation, and protect sperm cells from the immune system (blood-testis barrier) [7].

Sertoli cells recognise apoptotic germ cells through lipid membrane interactions, and seminolipids play a crucial role in spermatogenesis. Imaging mass spectrometry (IMS) locates lipids without labelling, allowing unknown molecule detection. In the testis, sulphated glycerogalactolipids dominate, with galactosylation catalysed by UDP-galactose:ceramide galactosyltransferase (CGT). CGT-deficient mice exhibit infertility, neurological abnormalities, and impaired spermatogenesis, highlighting the importance of sulfoglycolipids. Seminolipids stabilize sperm cell membranes and enable cell-cell adhesion, with affinity identified for 20 proteins. Phosphatidylcholines and polyunsaturated fatty acids (PUFAs) also play key roles in testicular structure and fertility [8,9,10,11,12,13].

The epididymis is divided into 19 segments or three regions (the caput, corpus, and cauda). Research has focused on tissue extracts and sperm samples, where MALDI imaging mass spectrometry was used to identify metabolites in the rat epididymis. The results showed distinct molecular clusters, each with specific lipid content, and it was found that during epididymal maturation, lipid content changed, with triacylglycerols decreasing and sphingolipids and plasmalogens increasing [14].

It has been recognised that lipids in testicles, including fatty acids, glycolipids, and seminolipids, have various biological functions. Concentrating on seminolipids, which are the main glycolipid constituents in mammalian testis and spermatozoa, it has been found that seminolipids are essential for spermatogenesis in male reproductive maturation. It was seen that total lipid content increases with age, and seminolipid presence may be related to spermatogenesis and sexual activity. That is the reason why identifying tissue biomarkers is crucial to assess testis health and fertility status [8,15,16,17].

Azoospermia, a pathological status where no sperm cell is present in semen, affects 10–15% of infertile men. This condition can be further categorised as obstructive or non-obstructive azoospermia. Sertoli cell-only syndrome (SCOS) is the most common pathological lesion in the background of non-obstructive azoospermia and is characterised by the complete absence of germinal epithelium, with Sertoli cells exclusively present in the seminiferous tubules. It is often caused by genetic disorders (e.g., Y chromosome microdeletion, Klinefelter syndrome) or environmental damage (e.g., toxin exposure, radiation, prior viral infection), but mostly idiopathic. In these cases, diagnostic tests include measurement of hormonal levels (elevated FSH levels are usually detected beside normal testosterone values), but definitive diagnosis is only provided by testicular biopsy, where testicular sperm extraction (TESE) is possible at the same time for assisted reproduction [18]. The treatment options vary depending on the severity of the condition. In milder cases, focal areas of partial/complete spermatogenesis can be found, these patients being able to have their natural offspring with assisted reproductive techniques (mostly using micro-TESE and intracytoplasmic sperm injection (ICSI)) [19,20,21,22].

The Johnsen scoring system is a histopathological classification that evaluates testicular damage and sperm maturity in testicular tubules on a scale of one to ten. It has been noted that scores above seven may indicate suitable sperm cells for artificial reproduction. This system assesses spermatogenesis, late spermatids, early spermatids, and germinal cells on the testicular tubules and derives a score based on those factors. The score ranges from ten, where full spermatogenesis is detected, to one, where no geminal epithelium can be found [23].

In order to understand the molecular assembly, it is critical to ascertain the types of molecules that are present on each cell. The localisation of lipids, which contain a broad diversity of molecular species based on their fatty acid content, using imaging mass spectrometry (IMS) was investigated. IMS offers a number of benefits for examining the two-dimensional distribution of lipids: firstly, it can be used to investigate localisation without the need for labels or specialised probes; secondly, it is a nontargeted imaging technique that enables one to find the localisation of unexpected metabolites; and thirdly, it can be used to simultaneously image multiple molecular species of metabolites. In this way, it can be demonstrated how significant alterations in lipid dynamics occurred throughout testicular development, based on IMS analysis of metabolites in mouse testis [8].

Notable previous research discovered how much opportunity lies within the matrix-assisted laser desorption/ionization imaging mass spectrometry (MALDI IMS) technique. The first piece of registered research (where the mouse animal model was examined) was carried out and published in 2006 by R. Lemaire et al. They found direct tissue analysis by MALDI-MS maintains tissue integrity, but spectral quality varies due to tissue type and freezing date. In this case, new matrices were tested, such as CHCA/2A4M5NP and CHCA/DANI, which offer better spectral quality, crystallisation, analysis duration, laser resistance, and fragmentation yield, enabling the use of MALDI IMS with high-frequency lasers and structural information [24]. The first paper about testis tissue investigations on mice by MALDI IMS was published in 2009 by N. Goto-Inoue. They found that seminolipid, a sulfated glyceroglycolipid, makes up 90% of glycolipids in mammalian testis, and furthermore, that the cell-specific localisation of seminolipids showed significant connections to testicular maturation [8]. Hashemitabar et al. discovered that asthenozoospermia, a common cause of male infertility, is characterised by reduced sperm motility. Researchers analysed sperm tail proteomics in normozoospermic and asthenozoospermic patients to identify novel biomarkers using the MALDI technique. It was demonstrated that these detected proteins may serve as markers for sperm cell dysfunction diagnosis, male contraception development, and embryo quality prediction [25]. Previously, Nielsen et al. reported heavy cannabis use may decrease semen quality by disrupting the endocannabinoid system (ECS) in the male reproductive tract. They found endocannabinoid-synthesising enzymes, diacylglycerol lipase and N-acyl-phosphatidylethanolamine-specific phospholipase D, as well as cannabinoid receptors, in testicular cells. As expected, the ECS was found to be involved in regulating testicular physiology, including spermatogenesis and Leydig cell function, suggesting that recreational use of cannabis may have adverse effects on testicular function [26]. MALDI IMS is a promising technique for investigating spatial arrangements of biomolecules such as lipids, peptides and proteins in tissue sections. It’s unsurprisingly popular in cancer research, but also has potential for molecular profiling and imaging of reproductive tissues. In line with expectations, recent publications related to embryo development, gene expression profiling, and biomarker discovery in reproductive system tumours. For these notable reasons, MALDI mass spectrometry came about to contribute to understanding reproductive system molecular mechanisms and pathological abnormalities [15].

In this paper, the spatial distributions of phospholipids have been investigated by MALDI IMS. Our results refer to the variation between normal spermatogenetic testis tissue and testis tissue with Sertoli cell-only syndrome (SCOS). In our study, we analysed local distribution of lipids in tissue sections from human testis by using MALDI IMS. Imaging mass spectrometry allows us to directly assign molecular information to tissue structures, thus our main goal is to identify and locally distribute biomarkers that could form the basis for a rapid diagnostic procedure, specific to the process of spermatogenesis.

## 2. Results

### MALDI Imaging Mass Spectrometry

**Figure 1:**
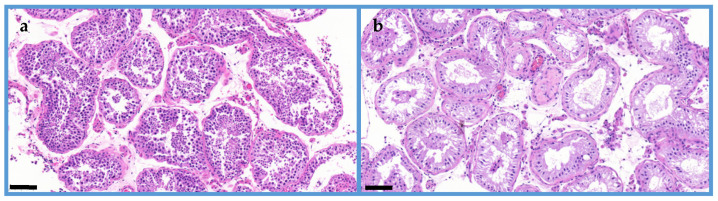
Histology of human testicular samples (**a**) Seminiferous tubule with complete spermatogenesis, many of type A and B spermatogonia; Johnsen score: 8–10. (**b**) Sertoli cell-only syndrome with normal adult and focal involuting Sertoli cells with triangular nuclei. The Sertoli cells nucleoli are prominent with cytoplasmic vacuoles. Johnsen score: 2. All scale bars: 100 μm.

In this study, human testicular phospholipid species were investigated by MALDI IMS. Spatial expression levels of the investigated lipid species are significantly altered in the normal and the SCOS individuals. Tissue cryosections of 12 azoospermic patients with Johnsen scores two to ten were investigated by MALDI IMS. Figure 1 illustrates the morphology of adult human testicular tissue in normal and SCOS conditions.

**Figure 2:**
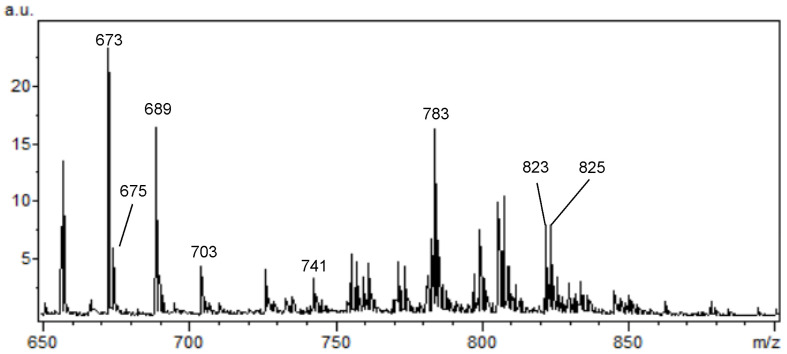
Summed positive ion MALDI TOF mass spectrum at the range of *m*/*z* 650 to 900 represents the typical spectral resolution and signal/noise ratio. The spectrum was recorded directly from the surface of the testis tissue cryosection with CHCA as matrix.

**Figure 3:**
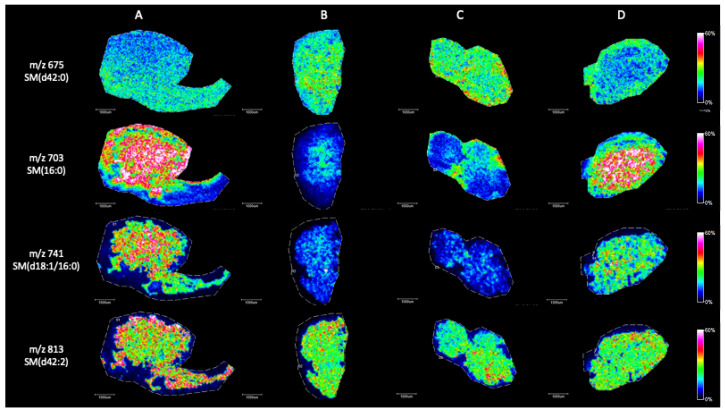
Representative sphingomyelin ion images (M+H^+^) of testicular tissue samples by MALDI IMS in positive mode with the use of CHCA as matrix. (**A**) Johnsen score 8–10, (**B**) Johnsen score 2, (**C**) Johnsen score 2, (**D**) Johnsen score 6–8. All scale bars: 1000 μm.

**Figure 4:**
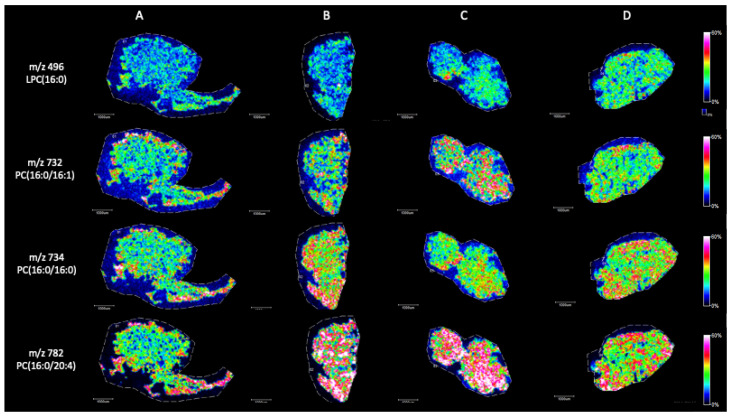
Representative palmitoyl lysophosphatidylcholine and phosphatidylcholine ion images (M+H^+^) of testicular tissue samples by MALDI IMS in positive mode with the use of CHCA as matrix. (**A**) Johnsen score 8–10, (**B**) Johnsen score 2, (**C**) Johnsen score 2, (**D**) Johnsen score 6–8. All scale bars: 1000 μm.

**Figure 5:**
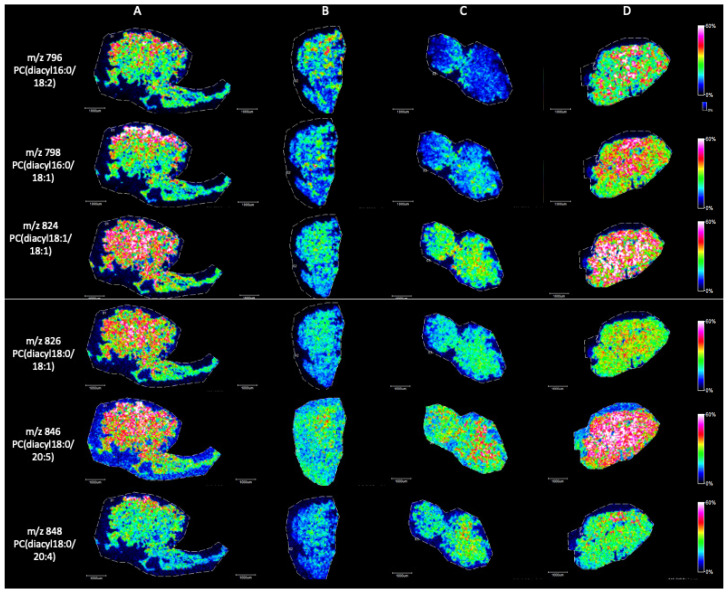
Representative ion images (M+H^+^) of phosphatidylcholine species with diacyl residues in testicular tissue samples by MALDI IMS in positive mode with the use of CHCA as matrix. (**A**) Johnsen score 8–10, (**B**) Johnsen score 2, (**C**) Johnsen score 2, (**D**) Johnsen score 6–8. All scale bars: 1000 μm.

**Figure 6:**
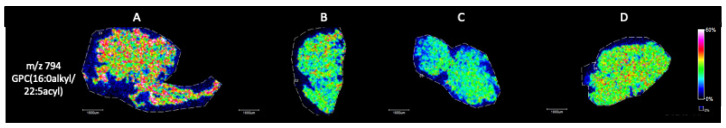
Representative glycerophosphatidylcholine ion images (M+H^+^) of testicular tissue samples by MALDI IMS in positive mode with the use of CHCA as matrix. (**A**) Johnsen score 8–10, (**B**) Johnsen score 2, (**C**) Johnsen score 2, (**D**) Johnsen score 6–8. All scale bars: 1000 μm.

**Figure 7:**
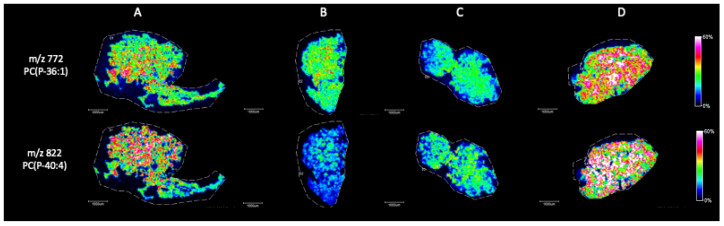
Representative plasmalogen ion images (M+H^+^) of testicular tissue samples by MALDI IMS in positive mode with the use of CHCA as matrix. (**A**) Johnsen score 8–10, (**B**) Johnsen score 2, (**C**) Johnsen score 2, (**D**) Johnsen score 6–8. All scale bars: 1000 μm.

**Figure 8:**
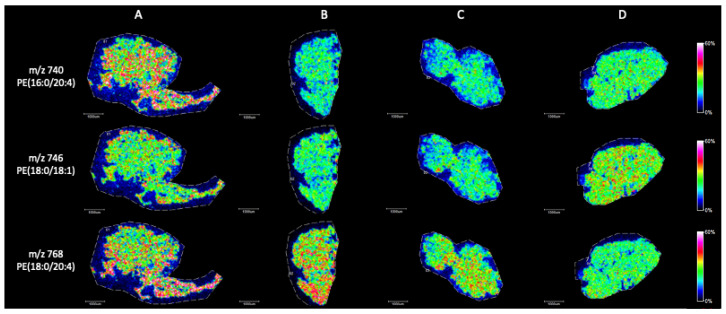
Representative phosphatidylethanolamine ion images (M+H^+^) of testicular tissue samples by MALDI IMS in positive mode with the use of CHCA as matrix. (**A**) Johnsen score 8–10, (**B**) Johnsen score 2, (**C**) Johnsen score 2, (**D**) Johnsen score 6–8. All scale bars: 1000 μm.

Briefly, in positive ion mode, we detected phosphatidylcholine (PC), sphingomyeline (SM) and phospatidylethanolamine (PE) peaks in the range of *m*/*z* 650 to *m*/*z* 900 (Figure 2). Figure 3, Figure 4, Figure 5, Figure 6, Figure 7 and Figure 8 represent the particular lipid maps of sphingomyelins, phosphatidylcholines, glycerophosphatidylcholine, plasmalogen and phosphatidylethanolamines respectively. The mass spectrometric images illustrate the protonated quasimolecular ions (M+H^+^) of lipid species, moreover, the sodium and potassium adducts (M+Na^+^, M+K^+^) of the identified lipids have been successfully detected in most cases.

Protonated ion images corresponding to SM (d42:0) *m*/*z* 675, SM (16:0) *m*/*z* 703, SM (d18:1/16:0) *m*/*z* 741 and SM (d42:2) *m*/*z* 813 are shown in Figure 3. The local distributions of sphingomyelins (16:0) and (d18:1/16:0) are significantly different in healthy (Johnsen score 8–10) and SCOS (Johnsen score 2) testis samples. The concentrations of SM (16:0) and (d18:1/16:0) are higher in samples with successful spermatogenesis and maturation. The level of SM containing very-long-chain polyunsaturated fatty acid (VLCPUFA), SM (d42:2) revealed a positive correlation with successful spermatogenesis, while SM (d42:0) slightly increased in SCOS samples.

The major phospholipid present in mammalian cells is PC, which accounts for ca. 50% of phospholipid pool [27]. In this study, various type of PCs have been detected with abundant peaks with positive ionization mode. Figure 4 shows selected (M+H^+^) ion images of palmitic acid containing phosphatidylcholines, such as LPC (16:0), PC (16:0/16:1), PC (16:0/16:0) and PC (16:0/20:4), respectively. In these cases, the concentrations of the lipid species showed higher levels in tissue sections with unsuccessful spermatogenesis (Johnsen score 2). The most significant differences can be found in the local distribution of 1-palmitoyl-2-arachidonoyl-sn-glycero-3-phosphocholine (PC (16:0/20:4), represented by *m*/*z* 782.

The local distributions of diacyl PCs show higher levels in normal samples, while the concentrations are significantly decreased in samples with lower Johnsen scores. The most spectacular alterations have been observed in the case of PC (16:0/18:2), PC (16:0/18:1), PC (18:1/18:1), and PC (18:0/20:5) (Figure 5).

The spatial distribution of glycerophosphatidylcholine (16:0-alkyl/22.5-acyl) shows detectable differences between the normal and the SCOS tissue sections. It was the highest level in the sample with a Johnsen score of 8–10, while the Johnsen score decreased in the other tissue sections (Figure 6).

Plasmalogens are unique ether lipids in spermatozoon membrane. Ether phospholipids are a type of glycerophospholipids with alkyl or alkenyl chains attached to the glycerol backbone [28]. They play a crucial role in physiological spermatogenesis, sperm maturation, maintenance of motility, membrane fluidity and lipid peroxidation. The concentration of plasmanyl-PC 40:4 has significantly decreased in SCOS conditions, while PC (P-36:1) slightly altered (Figure 7).

PE was detectable in the positive ionisation, however, it has lower sensitivity than PC or SM. Arachidonic acid containing PE species (16:0/20:4) and (18:0/20:4) were slightly reduced in SCOS samples (Figure 8) with homogenous spatial distributions.

**Figure 9:**
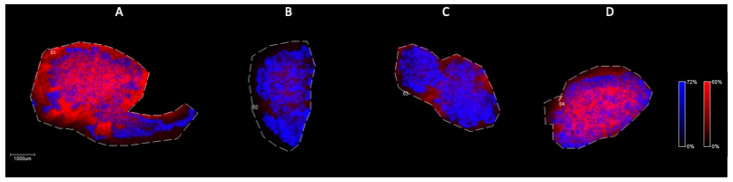
Merged ion images of SM (16:0) (*m*/*z* 703, red) and PC (16:0/20:4) (*m*/*z* 782, blue) in testicular tissue samples by MALDI IMS in positive mode with the use of CHCA as matrix. (**A**) Johnsen score 8–10, (**B**) Johnsen score 2, (**C**) Johnsen score 2, (**D**) Johnsen score 6–8. Scale bar: 1000 μm.

As, previously described, the levels of SM (16:0) were down-regulated in SCOS samples, in contrast the presence of PC (16:0/20:4). Figure 9 represents the different local distribution of these two lipid species in the testis cryosections.

## 3. Discussion

Azoospermia, defined as the absence of sperm cells in semen occurs in ca. 15% of the infertile male population. Previously, several analytical techniques have been used for lipidomic investigations of spermatozoa, semen, seminal plasma and testicular samples. These studies showed a significant correlation between successful spermatogenesis and male fertility with lipid compositions. MALDI IMS is a powerful method to determine the spatial distributions of molecular species, such as phospholipids. In this study, SM, PCs and plasmalogens were determined directly from azoospermic human testicular cryosections by MALDI TOF/TOF IMS. Our findings showed a strong relationship between the Johnsen scoring system and the expression levels of phospholipids. PC, PE and SM are the most abundant phospholipids in mammalian cells, as well as in human testis.

In mammalian cells, the glycerophophatidylcholines mainly occur in their diacyl form, additionally, alkenyl-acyl and alkyl-acyl species are also present [29]. Our results showed down-regulation of monounsaturated and polyunsaturated diacyl-PC species (16:0/18:2, 16:0/18:1, 18:1/18:1 and 18:0/20:5) in all SCOS testicular tissue samples, while the concentration of PC (16:0/20:4), PC (16:0/16:0) and PC (16:0/16:1) were overrepresented in azoospermic conditions.

Sphingomyelins are structural components of plasma membranes with a crucial importance in apoptosis, cell ageing, development, spermatogenesis, and sperm cell function. In physiological conditions of spermatogenesis, the concentration of SM (16:0) is significantly higher than in SCOS conditions, in contrast to the expression levels of some PC species. PCs are potential choline donors in SM biosynthesis, accordingly, the results may indicate the irregularity of SM synthetic pathways in SCOS conditions. Previous studies have also shown similar effects of the SM/PC ratio during epididymal maturation of sperm cells [29].

Plasmalogen are unique PCs in testis with strong antioxidative character, because the alkenyl ether bond is highly sensitive for reactive oxygen species (ROS). Their significant down-regulation shows increased oxidative nature and higher ROS concentration in SCOS testicular samples. [28,30].

Our preliminary MALDI IMS data collected from human testicular tissue cryosections showed a relationship between the Johnsen scoring system and the local distribution of phospholipid species.

## 4. Materials and Methods

### 4.1. Clinical Samples

In this study, human testicular tissue samples were used for investigations, gained via testicular sperm extraction (TESE) from azoospermic patients as part of infertility treatment. Altogether, 12 samples were examined: 3 with normal/slightly impaired spermatogenesis or maturation arrest (Johnsen score 6–10) and 9 with SCOS (Johnsen score 2), according to the results of conventional histopathological examination.

### 4.2. Histological Tissue Sections

For routine histological investigations, 4 µm thick FFPE sections (n = 5) were made by a Leica CM1860 UV cryostat (Biomarker Kft., Budapest, Hungary), the slides were stained with standard hematoxylin and eosin and documented by a Pannoramic Desk digital slide scanner (3D Histech, Budapest, Hungary), using Pannoramic Viewer 1.15 software for data evaluation.

### 4.3. MALDI Imaging Mass Spectrometry

Imaging mass spectrometry (IMS) makes it possible to combine the histological information with label-free mass spectrometric imaging technology.

Fresh testis tissue samples were stored at –80° C until processing. Freshly prepared 2% carboxymethyl cellulose embedding material was used for immobilisation, and a Leica CM1860 cryostat (Leica Microsystems GmbH, Wetzlar, Germany) was applied at –23 °C for tissue sectioning. Tissue was cut at a thickness of 15 μm and thaw-mounted onto indium-tin-oxide-coated glass slides (n = 5) (Bruker Daltonics, Bremen, Germany). Alpha-Cyano-4-hydroxycinnamic acid (CHCA, Bruker Daltonics, Bremen, Germany) matrix was applied in 50 deposition-drying cycles by using an automated piezoelectric spray device. The 7 mg/mL matrix solution was prepared fresh every day by dissolving CHCA in 6:4 acetonitrile—0.2% aqueous triluoroacetic acid (TFA, Spectranal quality, Sigma-Aldrich, Budapest, Hungary). Mass spectra were acquired on an Autoflex Speed MALDI TOF/TOF mass spectrometer equipped with a 1 kHz Smartbeam-II solid-state laser (Bruker Daltonics, Bremen, Germany). The MALDI measurements were performed in positive reflectron mode in a detection range of *m*/*z* 400 to 3000. The lateral resolution for MALDI imaging was set to 50 μm. A total of 300 laser shots were executed from each position. The acquisition and evaluation were carried out using the FlexImaging 3.0 and FlexControl 3.4 software (Bruker Daltonics, Bremen, Germany). Lipid identification was carried out by MALDI TOF/TOF mass spectrometry using LIFT mode for PSD (post-source decay) and CID (collision-induced decay) fragmentation.

## 5. Conclusions

This study, using MALDI IMS, is the first to demonstrate the local distribution of phospholipids, especially SM, PCs, and plasmalogens in human testicular samples with SCOS.

The aim of our research was to discover the correlation between the Johnsen scoring system and phospholipid expressions in testis of SCOS patients. In the future, based on our findings, the locally altered lipid species can be determined by a routine diagnostic technique during a microTESE procedure to make the possibility for successful sperm extraction easier. In case of NOA, the general success rate of a TESE procedure is only around 40%, because only focal spermatogenesis occurs. With these types of analyses, if we find any distinguishing mark between sperm-producing and non-sperm-producing regions of testis, the chance of finding sperm cells can be increased. This logic can also be transferred to other areas. Based on certain characteristics, it can allow the separation of sperm cells that carry the trait and those that do not.

## Data Availability

Data are contained within the article.
